# Cesarean Scar Endometriosis: An Uncommon Surgical Complication on the Rise? Case Report and Literature Review

**DOI:** 10.1155/2017/8062924

**Published:** 2017-02-23

**Authors:** Imane Khachani, Abdelhai Filali Adib, Rachid Bezad

**Affiliations:** Centre National de Santé Reproductrice, 1 rue Soekarno, 10100 Rabat, Morocco

## Abstract

Endometriosis is defined by the presence and growth of ectopic functional endometrial tissue outside the uterus. Scar endometriosis has been described following obstetrical and gynecological surgery. It is a rare condition, though probably on the rise, due to the considerable increase of cesarean sections performed worldwide. Its physiopathology is complex; its symptomatology is rich and diverse but thorough clinical examination along with ultrasound imaging and potentially pretherapeutic cytologic evaluation are usually efficient in diagnosing the condition. Treatment is mostly surgical. We report the case of a cesarean section scar endometriosis, managed at a tertiary level center and emphasize the diagnosis and treatment options.

## 1. Introduction

Endometriosis was first described by Karl Von Rokitansky in 1860. It is a chronic gynecologic disorder where the functional and morphological endometrial glands and stroma are present outside the uterine cavity [1]. It mainly affects women in reproductive ages. Major sites for extrapelvic endometriosis include the lungs, pleura, kidneys, bladder, omentum, bowel, lymph nodes, and abdominal wall.

Abdominal wall endometriosis is one of the most frequent extra pelvic locations, mostly occurring in old surgical scars from obstetrical and gynecological procedures [2]. The great variability of symptoms and clinical presentations as well as the limited knowledge on the disease can lead to diagnosis difficulties and delays that are detrimental to the patient's wellbeing and quality of life. We report the case of a cesarean section scar endometriosis (CSE) managed at the National Reference Center for Reproductive Health in Rabat and highlight the diagnosis steps and therapeutic management.

## 2. Case Presentation

A 37-year-old, G3P3, reported the appearance of a subcutaneous abdominal mass, with discreet menstrually related enlargement, pain, and sporadic brown leakage. Personal history revealed that she had undergone three lower segment cesarean sections; the two first ones were planned, respectively, at 39 and 38 weeks of gestational age for narrow pelvic outlet with uneventful postoperative period. The last one was an emergency, performed at 32 weeks for heavy placenta praevia hemorrhage, with positive fetal outcome. The mass appeared 7 months after the last cesarean section.

**Figure 1:**
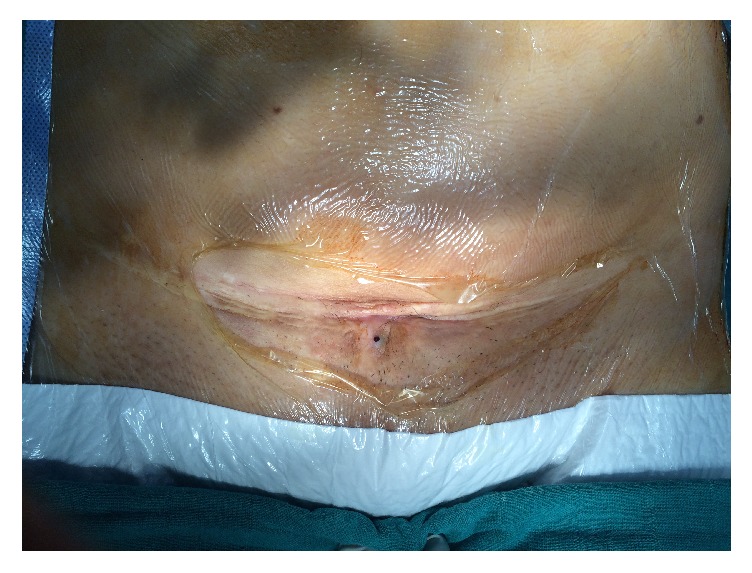
Cesarean section scar with subcutaneous mass and orifice with brown leakage.

**Figure 2:**
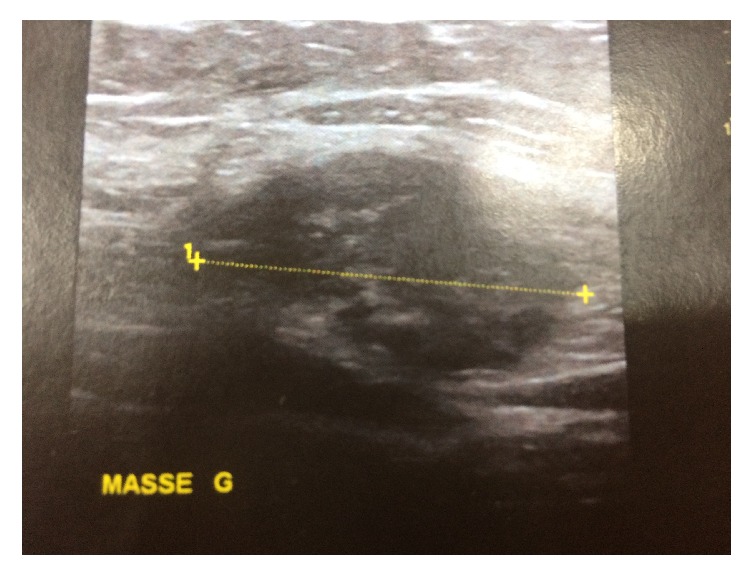
Heterogenous hypoechoic mass at sonography.

**Figure 3:**
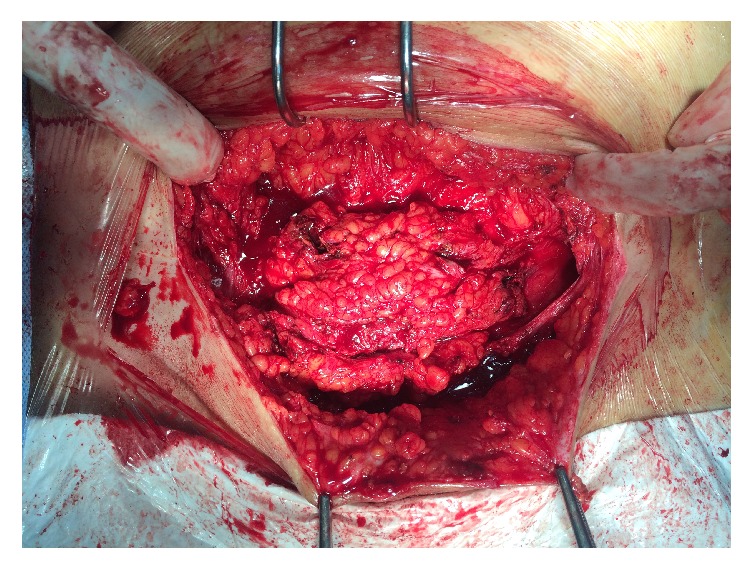
Surgical exploration showing mass margins and extensions into the fascia layer.

**Figure 4:**
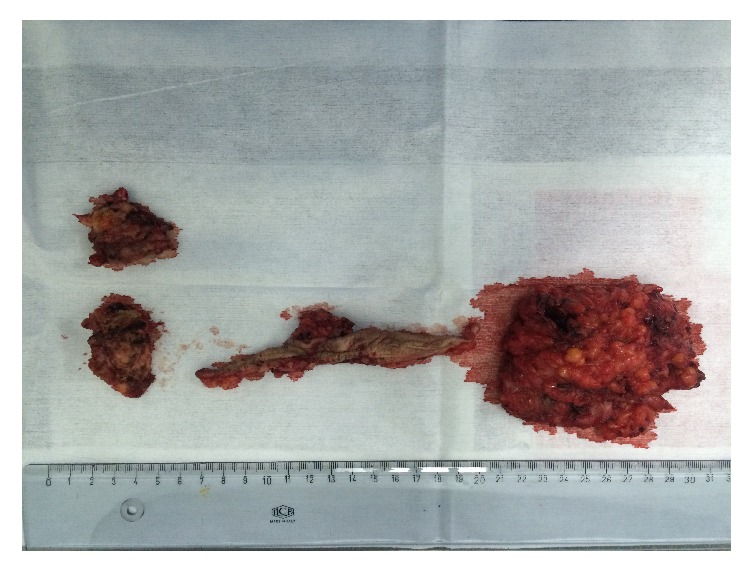
Final surgical outcome with mass and skin excision.

Physical examination performed during her menstruation period found a palpable tender subcutaneous oval mass of 80 × 35 mm, located under the incision scar, with a small 2 mm diameter skin orifice at the center of the mass, producing thick brown leakage when pressuring the skin on both sides of the orifice (Figure 1). Sonography and Doppler examinations of the abdominal wall soft tissue revealed a heterogeneous hypoechoic mass studded with echogenic spots. A few vascular satellite arterial structures were described (Figure 2). A complementary Computed Tomography reported the presence of a thin fistula between the mass and the skin and described its progression through the subcutaneous tissue. The clinical history, physical examination, and imaging features led to the diagnosis of CSE. Surgical wide en bloc excision was performed, ensuring surrounding macroscopic clear margins and including the covering skin and fistula orifice. The mass was surrounded by fibrosis and small extensions through the aponeurosis were found (Figure 3). They were thoroughly removed, and the subsequent defects were repaired after complete excision of the mass, with no need of mesh placement (Figure 4). The histological examination confirmed the presence of clusters of endometrial glands surrounded by endometrial-like stromal cells and haemosiderin-laden macrophages. The postoperative period was uneventful and periodic 6 months check-ups were set, with no recurrence observed during the 24 months following the surgery.

## 3. Discussion

Abdominal wall endometriosis is largely related to previous history of surgery [3]. Endometriosis implants developing in the subcutaneous tissue of surgical scars occur most frequently after gynecological and obstetrical procedures, including cesarean sections, hysterectomies, cystectomies, tubal ligations, and amniocenteses [4]. A few cases have been reported after episiotomy but remain far more rare [5, 6], and cases related to surgical scars of appendectomies, umbilical hernioplasties, and laparoscopic trocar tracts have also been described [7, 8]. It is interesting to note that Ideyi et al. and Chatzikokkinou et al. have published spontaneous cases, in absence of previous surgery, highlighting the complex multifactorial physiopathological processes behind the development of endometriosis [9, 10].

According to Nominato et al., cesarean section remains the most common surgical procedure related to the development of abdominal wall scar endometriosis [11]. They published an estimated incidence of 0.2 to 0.45% though it remains difficult to evaluate as most of the literature available is based on case reports and small series. More recently, in a study examining a cohort of 151 patients diagnosed with CSE, Zhang and Liu reported an incidence of 1.96% [12].

The pathogenesis of endometriosis is complex and CSE is believed to be the result of a mechanical iatrogenic implantation, through the direct inoculation of the abdominal fascia and/or subcutaneous tissue with endometrial cells during the surgical intervention, which, stimulated by estrogen, become active and expand [13]. Wang et al. examined the factors contributing to CSE and defined possible causes, including the easy separation and transport of endometrial cells by the amniotic fluid flowing into the pelvic cavity after hysterotomy; the large amount of endometrial cells liberated into the pelvis before hysterotomy closure and that can potentially be trapped in the wound; and the nurturing role of blood and hormones, after inoculation of the cells, allowing them to grow and develop into subcutaneous masses [14]. It is important to highlight that higher incidence is reported after early hysterotomy (end of second or beginning of third trimester), as early decidua seems to have more pluripotential capabilities and can result in enhanced cellular replication producing endometriosis [15]. This was supported by our case, as the patient developed CSE after a cesarean at 32 weeks.

The presence of hormone-sensitive tissue under the skin explains the symptoms reported by our patient, including cyclic pain, swelling, and the very evocative blood-like brown leakage during menstruations. Pain—either cyclical or noncyclical—remained the major symptom, reported by more than 80% of patients in the cohorts of Zhang and Liu in China, Uçar et al. in Turkey, and Vellido-Cotelo et al. in Spain [12, 16, 17]. A mass was present at examination of more than 70% of patients in these studies. With regard to imaging, ultrasound is the most accessible, reliable, and cost-effective imaging technique for the diagnosis of CSE according to Hensen et al. [18], allowing—along with clinical examination—a differential diagnosis with incisional hernia, hematoma, abscess, cyst, or lipoma in most cases. In the study of Zhang and Liu, it also revealed deep infiltrations in 26% of patients, which helped guide the surgical excision [12]. Computed Tomography or Magnetic Resonance Imaging can be used in case of diagnosis doubt but they are rarely needed. Uçar et al. found no evidence of pelvic endometriosis associated for the 12 cases of CSE examined in their study [16]. Vellido-Costelo et al. highlighted that there seem to be no linkages between pelvic and scar endometriosis development. In their study, 14% of patients had associated pelvic endometriosis, which corresponds to the incidence in the general population [17].

Based on their clinical experience of fine needle aspiration cytology (FNAC) for 9 cases of CSE, Medeiros et al. have argued that it is a quick, cost-effective, and accurate diagnosis tool to include in patients' management [19]. They encouraged the use of this technique to provide a histopathological diagnosis prior to the surgical procedure in cases of uncertainty on the origin of the mass. In the study of Vellido-Cotelo et al., 52% had a FNAC diagnosis before surgery and one of the patients was diagnosed with cancer by this method, which subsequently led to a different therapeutic management [17]. However, the use of this technique is controversial, as some authors have warned against an increased risk of producing new endometriotic implants at the puncture site, as well as viscera injury if the diagnosis is uncertain [6]. In the case of our patient, clinical examination patterns and imaging features were sufficient to suggest the diagnosis of CSE and there was no need to perform FNAC.

Therapeutic management is essentially based on large surgical excision, with clear margins and reconstruction of damaged tissue. Medical treatment involving hormone suppression has been suggested to relieve clinical symptoms [20] but, according to Al-Jabri, it only gives partial relief and recurrence after the cessation of medication is constant [21]. Recurrence rates after surgery are variable but seem generally low. No recurrence was reported by Uçar et al. for a follow-up period ranging from 12 to 60 months [16]. Horton et al. found a recurrence rate of 4.3% [7], while Zhang and Liu reported a recurrence of 7.8% over an average period of 20 (±16) months [12]. Most authors agreed that surgery is effective in preventing recurrence, as well as conversion to malignancy, which—although quite rare—has been described in a few sporadic cases [22, 23].

The role of prevention has been explored in several studies. Picod et al. recommended thorough isolation of the surgical incision site and abundant lavage of the pelvic cavity with saline before closure of the wall [24]. Other studies suggested that absence of closure of the parietal and visceral peritoneum may significantly increase the risk of endometriosis in the skin incision scar [25]. Lastly, instrument and needle replacement when suturing more superficial abdominal layers was also advanced, in order to avoid iatrogenic inoculation of endometrial cells [26]. These recommendations were all based on the various physiopathological hypotheses suggested for the constitution of CSE. No studies have explored them and further research is needed to establish effective measures to prevent abdominal wall postsurgical endometriosis.
